# Supplementary material for: Pre-analytical error for three point of care venous blood testing platforms in acute ambulatory settings: A mixed methods service evaluation
Source: PLoS One. 2020 Feb 3;15(2):e0228687. doi: 10.1371/journal.pone.0228687 (PMC6996845; doi:10.1371/journal.pone.0228687)
Supplement: S1 Appendix — (DOCX) [file pone.0228687.s001.docx]

**Supporting information 1 : Relevant i-STAT quality check failure codes**

*Groupings used to record errors up to February 2017*

| **Code** | **Description** | **Likely user error** |
| --- | --- | --- |
| 1 | Other |  |
| 2 | Environment |  |
| 3 | Cartridge Handling |  |
| 4 | Overfilled Cartridge | ★ |
| 5 | Unable to Position Sample |  |
| 6 | Underfilled Cartridge | ★ |
| 7 | Insufficient Sample | ★ |
| 8 | Thermal Contact |  |

*i-STAT failure codes recorded from February 2017*

| **Category/ Code** | **Description** | **Likely user error** |
| --- | --- | --- |
| **Battery error** |  |  |
| 1 | Dead batteries: there is insufficient power to complete the testing cycle |  |
| **Barcode error** |  |  |
| 15 | Barcode does not match cartridge type |  |
| **Coagulation error** |  |  |
| 19 | No clot detected during the coagulation test cycle |  |
| 22  25 | Cartridge error: for coagulation cartridges, the mixing of the sample and the reagent is compromised; caused by an insufficient or clotted sample, or by air bubbles in the sample |  |
| 26 | Cartridge error: a coagulation specific quality check failure; premature substrate activation, abnormally low levels of substrate, or invalid fluid motion |  |
| **Cartridge error** |  |  |
| 20  27  29  33  41  45 | Cartridge error, such as calibrant fluid arriving too soon, too late, or not at all, or noise in the calibrant fluid signals; can be caused by poor contact |  |
| 21 | Cartridge preburst: the analyzer detected fluid on the sensors before it should have; causes are poor metalization of the chips, dirt on the metalization, or bent or broken thermal proves in the analyzer |  |
| 24 | Cartridge error: the electrical resistance of the calibrant fluid used to verify the electrolyte concentration is out of specification |  |
| 31  34  44 | Unable to position sample: the analyzer did not detect movement of sample across the sensors |  |
| 43 | Cartridge error: the amperometric sensor was out of specification |  |
| 46 | Cartridge error: the analyzer did not detect movement of sample across the sensors |  |
| 69 | Cartridge type not recognised: when running coagulation cartridges, may be caused by poor contact between the analyzer pins and the cartridge |  |
| **Contact error** |  |  |
| 23 | Poor contact detected between the analyzer contact pins and the cartridge sensor contact pads: may be corrected by conditioning the analyzer contact pins |  |
| 49 | Poor contact detected between the analyzer contact pins and the cartridge identification chip contact pads: may be corrected by conditioning the analyzer contact pins |  |
| 79  80  81 | Cartridge error: bad contact between the thermal probes in the analyzer and metalization on the back of the chips in the cartridge, caused by poor metalization, dirt, or bent or broken thermal probes |  |
| **Overfill** |  |  |
| 30 | Sample positioned beyond fill mark: cartridge overfilled | ★ |
| 127 | Cartridge error: a wet sensor was detected before the initial sample movement; possible overfilled or used cartridge | ★ |
| 146 | Cartridge error: overfilled cartridge | ★ |
| **Underfill** |  |  |
| 35  36 | Sample positioned short of fill mark: cartridge underfilled | ★ |
| 145 | Cartridge error: analyzer failed to detect fluid arrival upon the initial sample push; may be caused by a cartridge leak, failure to close the cartridge completely, or underfilled cartridge | ★ |
| **Insufficient sample** |  |  |
| 38  39 | Insufficient sample: insufficient sample in the sample well, or bubbles in the sample | ★ |
| **Insertion error** |  |  |
| 47 | Cartridge not inserted properly | ★ |
| **Analyzer/ motor error** |  |  |
| 50 | Analyzer error: the motor has moved too far |  |
| 52 | Analyzer error: the motor stalled while moving |  |
| 56 | Analyzer error: mechanical or electronic failure; noise on the thermal circuit; may be the result of electronic interference |  |
| 66  85 | Analyzer error: mechanical or electronic failure |  |
| **Immunoassay – analysis fluid** |  |  |
| 120  121  122  124  133  148 | Cartridge error: a problem with the movement of the analysis fluid during the cartridge run |  |
| 129  142 | Cartridge error: analysis fluid mixed with the sample |  |
| **Immunoassay – error in QC run** |  |  |
| 123 | Cartridge error: the quality control during the cartridge run failed to verify the presence of active immuno reagents |  |
| 126 | Cartridge error: the quality control during the cartridge run failed to verify the integrity of the analysis fluid; may also be related to poor electrical connection between the i-STAT 1 Analyzer and the cartridge |  |
| **Immunoassay – poor filling** |  |  |
| 128  131  132  134  135  136  137 | Cartridge error: most often related to poor filling of an immunoassay cartridge, the presence of sample bubbles, or the abrupt insertion of a cartridge into the analyzer | ★ |
| **Immunoassay – atypical data stream** |  |  |
| 149  150  151 | Cartridge error: the analyzer detected an atypical data stream from the cartridge |  |
| **Field length** |  |  |
| MT_2020 | Field length exceeded |  |

**Supporting information 2 : Relevant i-STAT Alinity quality check failure codes**

| **Code** | **Description** | **Likely user error** |
| --- | --- | --- |
| **Cause 3 codes** | Cartridge was rejected during the testing cycle. Probable causes: operator pressed too hard on the centre of the cartridge; used cartridge inserted; cartridge was frozen and thawed before testing |  |
| 21-01-3.1.3  23-01-3.3.2 | Repeat testing with a freshly filled cartridge | ★ |
| **Cause 6 codes** | Excess blood was added to the cartridge |  |
| 30-02-4.1.4 | When filling a cartridge, use care to advance blood to the level indicated by the ‘fill to’ arrow | ★ |
| **Cause 7 codes** | Sample was rejected during the testing cycle. Probable cause: snap closure not secure |  |
| 31-01-4.1.5  34-01-4.1.11 | Repeat testing with a freshly filled cartridge | ★ |
| **Cause 8 codes** | An insufficient amount of blood was used to fill the cartridge |  |
| 35-01-4.1.7  36-01-4.1.10 | When filling a cartridge, use care to advance blood to the level indicated by the ‘fill to’ arrow | ★ |
| **Cause 9 codes** | Sample was rejected during the testing cycle. Probable causes: bubbles in the sample; insufficient amount of sample used to fill the cartridge |  |
| 38-01-4.1.12 | Repeat testing with a freshly filled cartridge | ★ |
| **Cause 15 codes** | Cartridge was rejected. Probable causes: instrument cannot lock cartridge in place to begin testing; debris on cartridge |  |
| 79-01-2.3.1 | Power off the instrument. Repeat testing with a freshly filled cartridge |  |
| **Codes of unspecified cause** |  |  |
| 27-01-4.1.1 | Repeat testing with a freshly filled cartridge |  |
| 32-01-4.1.6 | Repeat testing with a freshly filled cartridge |  |
| 47-01-2.1.7 | The cartridge was not fully inserted. Repeat testing with a freshly filled cartridge |  |
| 69-02-4.6.4 | Always scan the barcode found on the pouch that contained the cartridge in use. Repeat testing with a freshly filled cartridge |  |
| 80-01-3.4.4 | Repeat testing with a freshly filled cartridge |  |
| 95-01-0.0.0 | Test has been successfully cancelled |  |
| **Process error codes** |  |  |
| 90-02-2.4.14  90-02-2.4.15  90-02-2.4.16 | Perform an Electronic Simulator Test |  |

Some descriptions have been shortened for clarity. Reasons for error are given for error code groupings, where this is specified in the operations manual. Suggested resolutions are provided for certain error codes.

**Supporting information 3 : Relevant Afinion AS100 and Afinion 2 codes**

| **Code** | **Description** | **Likely user error** |
| --- | --- | --- |
| **Codes caused by assay specific limitations** |  |  |
| 101 | Hematocrit too low (Afinion 2 only) |  |
| 102 | Hematocrit too high (Afinion 2 only) |  |
| **Codes caused by sample or test cartridge failure** |  |  |
| 201 | Insufficient sample volume (empty capillary; air bubble in capillary; capillary incompletely filled) | ★ |
| 202 | Excess sample on the sampling device exterior | ★ |
| 203 | Wrong sample material | ★ |
| 204 | Coagulated sample; hemolysed blood sample (or poor sample quality – Afinion 2 only); test cartridge or analyzer failure | ★ |
| 208 | Test cartridge previously used | ★ |
| 210 | Test cartridge temperature too low |  |
| 212 | Test cartridge not recognised by the analyzer (or software upgrade is required – Afinion 2 only) |  |
| 213, 214 | Test cartridge or analyzer failure |  |
| 215 | Test cartridge or analyzer failure (or haemolysed blood sample or poor sample quality – Afinion 2 only) |  |
| **Codes caused by analyzer failure** |  |  |
| 302 | Analyzer failure |  |
| 304 | Analyzer temperature is too low |  |
| **Other information codes (process errors)** |  |  |
| 404 | Operator ID is not found in operator list |  |
| MT_16002 | Barcode operator ID error |  |

Some descriptions have been shortened for clarity. Error codes where the description in the Afinion 2 manual differs slightly from that in the Afinion AS100 manual are indicated in the table above.
