# Supplementary material for: Pre-analytical error for three point of care venous blood testing platforms in acute ambulatory settings: A mixed methods service evaluation
Source: PLoS One. 2020 Feb 3;15(2):e0228687. doi: 10.1371/journal.pone.0228687 (PMC6996845; doi:10.1371/journal.pone.0228687)
Supplement: Attachment — Submitted filename: Response to reviewers.docx [file pone.0228687.s004.docx]

**Response to reviewers**

Editor:

In addition to the few issues raised by the reviewers, please reconsider revising the title to clearly state that the paper is on the effect of pre-analytic handling on three POCT instrument errors.

We agree and have modified the title to reflect this:

Pre-analytical error for three point of care venous blood testing platforms in acute ambulatory settings : a mixed methods service evaluation

One of the most common pro-analytic errors insufficient patient ID.

This was true of the Afinion but this was likely to be only a technical result in accessing the device (quickly resolved by the operator) and so not likely to be an operator error. As such, although we thought it appropriate to report this in results tables we do not feel that it warrants more prominence than currently.

Reviewer #1: The study consider the pre—analytical errors recorded with the use of three specific devices, both in an hospital setting and community in ambulatory care. The authors consider the errors registered by the instruments and in particular the errors attributable to the operators.

The errors in the pre-analytical phase include many aspects, in addition to those that can be registered by the analytical system: the choice of the right timing with respect to the clinical conditions (for example for blood gas, you could quote Auvet doi: 10.1186/s13613-016-0152-6 ), the site of the sampling, the instruments used for venipuncture, the any dilution or contamination of the blood drawn, the sample hemolysis. Despite this, the study provides new data on the safety of POCT systems. This limitation must be discussed.

Thank you – we agree that this should be mentioned and we have added to the relevant part of the Discussion: “It was not possible to assess whether an incorrectly filled cartridge or poor quality sample might have given an inaccurate and therefore clinically misleading test result if the device did not initiate an error message, and this may be affected by the site of sampling or the timing when the test was performed [28]” (citing the paper suggested).

Another limitation of the study consists in the typology of errors that are strictly related to the specific type of instrument considered. These results cannot be reported to other types of analyzers.

We have added a sentence to the limitations in the Discussion: “Our results only relate to the POCT devices already used in the clinical environment considered in our study, and cannot be extrapolated to other analyzers.”

In the qualitative study the secondary analysis is based on limited number of cases.

The "Staff awareness of factors", “staff actions” and “staff perception” chapters (lines 329 - 512) present individual anecdotal cases that could be placed in a section of additional materials rather than in the text of the paper. Alternatively, the proposed reports could be summarized.

In qualitative research, emphasis is placed on gaining rich, deep information from a relatively small number of cases, where each has substantial experience of, and involvement in, the phenomenon under investigation (in this case, POC testing). The secondary analysis strand is based on data collected in a qualitative case study of one ambulatory care unit published elsewhere (Jones C, Glogowska M, Locock L, Lasserson D. Embedding new technologies in practice – a normalization process theory study of point of care testing. BMC Health Surv Res. 2016;16:591) which encompassed 14 episodes of ethnographic observation (approximately 60 hours) and 7 semi-structured interviews with staff (6 of which were audio-recorded and transcribed). In a similar way, the prospective ethnographic strand (which took place in another ambulatory care unit), represented 15 hours of observation.

For a qualitative study, this represents a considerable dataset, which was able to provide both a description of the context in which the POC testing had been implemented and a detailed interpretation of how usual practice around POC testing took place.

As regards the “Staff awareness of factors”, “staff actions” and “staff perception” sections, the material here are rigorously collected and robustly analysed data from the secondary analysis and the prospective ethnographic strands of the qualitative component. The qualitative component was part of the original mixed methods design which aimed at producing a more comprehensive assessment of preanalytical error in POC testing in this setting. Where quotations are included, it is because they are reflective of the views of more than just the one participant to whom they are attributed and are not simply ‘anecdotal cases’. Thus, we regard the qualitative material as playing an important role in aiding our understanding, and providing some explanation of, the quantitative findings and do not think it should be moved into additional materials.

In the conclusions a positive judgment is expressed regarding the frequency of errors attributable to the operators. But it is not known what the impact of these errors on care was, as the authors themselves claim, therefore the statement appears arbitrary and must be more prudent. In absolute terms the frequency is actually high, for example if we estimate it with the sigma metric.

We agree that our study was unable to assess the impact on patient care (this was not the objective). We have amended the Conclusions of the Abstract as a result: “In the observed settings, the rate of errors attributable to operators of the primary point of care device was less than 1 in 40. In some cases, errors may lead to a small increase in resource use or time required so adequate staff training is necessary to prevent adverse impact on patient care.”

Figure 3 shows little relevant data that can be briefly presented in the text.

We have moved this figure to a Supporting Information file (S2 Graph) and shortened the text.

Reviewer #2: This is a well written manuscript with focus on a topic for which there are very few published studies.

I only have one comment. The authors found that there was no difference in error rates between locations performing high and low volume POCT. I would have expected the error rate to be lower in areas performing high volume testing. Could the authors speculate or comment on why they saw no difference?

Thank you – we had also anticipated that the error rate might be lower in areas performing high volume testing but were limited in the amount of location-specific information that could be collected to determine factors that might influence error rates at different locations. We have amended the relevant part of the Discussion to now read: “Error rates in different locations did not appear to be strongly associated with total test use. This was unexpected but could be explained by location specific factors that we were unable to measure, such as the experience of individual operators. An interesting extra research question would be to investigate whether new operators have higher error rates or improve with practice.”
